# Supplementary material for: Retinoblastoma Survival Following Primary Enucleation by AJCC Staging
Source: Cancers (Basel). 2021 Dec 13;13(24):6240. doi: 10.3390/cancers13246240 (PMC8699512; doi:10.3390/cancers13246240)
Supplement: Supplementary file 1 [file cancers-13-06240-s001.zip › cancers-1466952-supplementary.pdf]

ID	Sex	Laterality	FU (months)	Age at Dx	IIRC	cTNM	Dx to Enu	pTNM 8th	Post-enu chemo (cycles)	OS	DSS	Dx to death (months)
1	M	OD	108	14	E	3d	0.2	pT3d	6	Neg	Neg	
2	M	OS	81	23	D	2b	0.2	pT1	0	Neg	Neg	
3	M	OS	110	20	E	3b	0.1	pT3b	6	Neg	Neg	
4	M	OS	77	53	D	2b	0.2	pT1	0	Neg	Neg	
5	F	OD	98	21	D	2b	0.0	pT1	0	Neg	Neg	
6	F	OD	60	35	E	3d	0.2	pT1	12	Neg	Neg	
7	F	OD	94	1	E	3d	0.0	pT1	0	Neg	Neg	
8	M	OD	91	26	E	3c	0.0	pT1	0	Neg	Neg	
9	M	OD	92	22	D	2b	0.1	pT1	0	Neg	Neg	
10	F	OD	29	19	?	?	0.0	N/A	0	Neg	Neg	
11	F	OS	82	36	E	3b	0.0	pT1	0	Neg	Neg	
12	M	OS	58	13	E	3d	0.0	pT1	0	Neg	Neg	
13	M	OD	72	60	?	?	0.0	N/A	0	Neg	Neg	
14	M	OD	99	12	D	2b	0.0	pT1	0	Neg	Neg	
15	M	OD	87	17	E	3d	0.2	pT3b	3	Neg	Neg	
16	M	OD	127	26	D	2b	0.1	pT1	0	Neg	Neg	
17	M	OD	98	2	E	3c	0.0	pT1	6	Neg	Neg	
18	M	OS	59	36	E	3b	0.1	pT1	0	Neg	Neg	
19	M	OD	140	73	E	3c	0.0	pT1	2	Neg	Neg	
20	F	OS	113	61	E	3b	10.1	pT1	0	Neg	Neg	
21	F	OD	108	30	E	3c	0.2	pT3b	6	Neg	Neg	
22	M	OS	94	21	E	3b	0.0	pT1	0	Neg	Neg	
23	M	OD	81	23	E	3c	0.0	pT1	0	Neg	Neg	
24	F	OS	97	25	E	3b	0.0	pT2b	0	Neg	Neg	
25	F	OS	116	24	E	3b	0.2	pT1	0	Neg	Neg	
26	M	OS	126	26	E	3b	0.1	pT1	0	Neg	Neg	
27	M	OD	104	62	E	3b	0.0	pT1	0	Neg	Neg	
28	F	OD	46	49	E	3d	0.3	pT3b	2	Neg	Neg	
29	M	OS	60	20	E	3d	0.0	pT1	0	Neg	Neg	
30	F	OS	140	7	E	3b	0.3	pT3b	3	Neg	Neg	
31	M	OS	120	42	E	3b	0.0	pT1	0	Neg	Neg	
32	M	OS	8	22	E	3d	0.2	pT4	6	Pos	Pos	8
33	M	OS	155	26	E	3b	1.0	pT3b	9	Neg	Neg	
34	M	OD	58	75	D	2b	0.0	pT2a	6	Neg	Neg	
35	F	OS	16	21	E	3c	0.0	pT1	0	Pos	Pos	16
36	M	OD	115	29	E	3c	0.0	pT1	0	Neg	Neg	
37	M	OS	97	8	E	3b	4.0	pT1	3	Neg	Neg	
38	F	OD	89	45	E	3b	0.0	pT1	0	Neg	Neg	
39	F	OD	89	24	D	2b	0.0	pT1	0	Neg	Neg	
40	F	OS	112	17	D	2b	0.2	pT1	0	Neg	Neg	
41	F	OD	142	34	E	3b	0.4	pT1	2	Neg	Neg	
42	F	OS	96	14	E	3d	0.0	pT3b	6	Neg	Neg	
43	F	OD	99	67	E	3b	0.0	pT1	0	Neg	Neg	
44	M	OD	12	55	E	3c	0.0	pT1	0	Pos	Pos	12
45	F	OS	77	27	E	3c	0.0	pT2a	3	Neg	Neg	
46	M	OD	57	33	E	3b	0.7	pT1	2	Neg	Neg	
47	M	OS	104	30	E	3c	0.2	pT2b	0	Neg	Neg	
48	M	OD	76	5	E	3b	0.0	pT1	0	Neg	Neg	
49	M	OD	51	28	E	3b	0.4	pT3b	3	Neg	Neg	
50	M	OS	17	77	E	3b	1.0	N/A	0	Neg	Neg	
51	M	OS	71	10	E	3c	0.4	pT1	0	Neg	Neg	
52	M	OD	13	25	E	3c	0.2	pT1	0	Neg	Neg	
53	M	OS	84	5	E	3b	0.0	pT1	0	Neg	Neg	
54	F	OD	59	33	D	2b	0.0	pT3b	6	Neg	Neg	
55	M	OD	72	3	D	2b	0.2	pT1	0	Neg	Neg	
56	M	OS	52	27	D	2b	0.1	pT1	0	Neg	Neg	
57	F	OS	79	2	?	?	0.0	N/A	0	Neg	Neg	
58	M	OD	138	21	D	2b	0.0	pT1	3	Neg	Neg	
59	M	OD	110	9	D	2b	0.2	pT1	0	Neg	Neg	
60	M	OD	89	36	E	3b	0.0	pT3b	6	Neg	Neg	

ID	Sex	Laterality	FU (months)	Age at Dx	IIRC	cTNM	Dx to Enu	pTNM 8th	Post-enu chemo (cycles)	OS	DSS	Dx to death (months)
61	M	OS	5	36	E	3d	0.0	pT3b	3	Pos	Pos	5
62	M	OD	49	29	E	3b	0.2	pT1	0	Neg	Neg	
63	M	OS	60	15	D	2b	0.0	pT1	0	Neg	Neg	
64	F	OS	110	4	E	3d	0.0	pT3b	6	Neg	Neg	
65	M	OD	130	17	D	2b	0.1	pT1	0	Neg	Neg	
66	F	OD	126	6	D	2b	0.1	pT3b	6	Neg	Neg	
67	F	OS	92	28	E	3b	0.0	pT3b	6	Neg	Neg	
68	F	OD	105	83	D	2b	0.1	pT1	0	Neg	Neg	
69	F	OS	97	26	E	3c	0.1	pT3b	6	Neg	Neg	
70	M	OD	119	24	D	2b	0.1	pT1	0	Neg	Neg	
71	F	OD	103	21	E	3b	0.3	pT3b	6	Neg	Neg	
72	F	OS	92	13	E	3c	0.1	pT3b	6	Neg	Neg	
73	F	OD	84	39	D	2b	0.0	pT1	0	Neg	Neg	
74	M	OS	137	18	E	3c	0.0	pT3b	4	Neg	Neg	
75	M	OD	122	22	D	2b	1.0	pT3b	6	Neg	Neg	
76	M	OD	69	28	E	3c	0.1	pT1	0	Neg	Neg	
77	M	OS	56	26	E	3d	0.1	N/A	0	Neg	Neg	
78	M	OD	99	67	D	2b	0.2	pT1	0	Neg	Neg	
79	F	OS	142	20	E	3c	0.2	pT1	3	Neg	Neg	
80	M	OD	122	18	D	2b	0.2	pT1	0	Neg	Neg	
81	F	OS	85	20	E	3c	0.1	pT3b	6	Neg	Neg	
82	M	OS	10	29	E	3c	0.0	pT3b	4	Pos	Pos	10
83	F	OS	72	24	E	3d	0.2	pT3b	6	Neg	Neg	
84	F	OD	73	14	E	3b	0.0	pT1	3	Neg	Neg	
85	M	OD	126	75	E	3c	0.0	pT3b	6	Neg	Neg	
86	F	OS	100	51	E	3b	0.0	pT2b	4	Neg	Neg	
87	F	OD	91	9	D	2b	0.0	pT3a	4	Neg	Neg	
88	M	OS	116	19	D	2b	0.0	pT1	1	Neg	Neg	
89	M	OS	88	28	?	?	0.0	N/A	0	Neg	Neg	
90	M	OD	123	12	D	2b	0.0	pT1	0	Neg	Neg	
91	F	OS	65	25	E	3d	0.0	pT1	0	Neg	Neg	
92	M	OD	75	19	E	3d	0.0	pT3d	5	Neg	Neg	
93	M	OS	83	20	E	3c	0.0	N/A	0	Neg	Neg	
94	M	OS	40	25	D	2b	0.1	pT1	0	Neg	Neg	
95	F	OD	88	23	D	2b	0.0	pT1	4	Neg	Neg	
96	M	OS	127	38	D	2b	0.1	pT1	0	Neg	Neg	
97	M	OD	52	20	D	2b	0.2	pT1	0	Neg	Neg	
98	F	OD	78	57	D	2b	0.3	pT1	0	Neg	Neg	
99	F	OD	159	22	E	3b	0.0	pT3b	9	Neg	Neg	
100	F	OS	98	23	E	3b	0.0	pT1	0	Neg	Neg	
101	M	OD	104	20	E	3b	0.3	pT3b	3	Neg	Neg	
102	F	OS	86	2	E	3b	0.0	pT1	0	Neg	Neg	
103	M	OS	69	29	D	2b	0.0	pT1	0	Neg	Neg	
104	F	OD	97	18	E	3d	0.0	pT1	3	Neg	Neg	
105	F	OD	157	18	?	?	0.0	N/A	0	Neg	Neg	
106	M	OD	67	21	E	3b	0.0	pT1	6	Neg	Neg	
107	F	OS	15	32	D	2b	0.0	pT1	3	Neg	Neg	
108	F	OS	124	42	D	2b	0.1	pT1	0	Neg	Neg	
109	F	OD	61	24	D	2b	0.0	pT1	0	Neg	Neg	
110	M	OD	90	35	D	2b	0.1	pT1	1	Neg	Neg	
111	M	OS	122	3	E	3a	12.8	pT1	0	Neg	Neg	
112	M	OD	151	57	D	2b	0.0	pT1	3	Neg	Neg	
113	F	OD	77	35	D	2b	0.2	pT1	0	Neg	Neg	
114	M	OS	86	16	E	3d	0.2	pT3b	4	Neg	Neg	
115	M	OS	74	22	D	2b	0.2	pT1	0	Neg	Neg	
116	M	OD	35	50	?	?	0.0	N/A	13	Pos	Pos	35
117	M	OD	122	24	E	3b	0.0	pT3b	6	Neg	Neg	
118	M	OS	19	18	E	3c	0.0	pT3b	6	Neg	Neg	
119	F	OD	138	27	E	3d	0.0	pT3b	6	Neg	Neg	
120	F	OD	79	4	?	?	0.0	N/A	0	Neg	Neg	

ID	Sex	Laterality	FU (months)	Age at Dx	IIRC	cTNM	Dx to Enu	pTNM 8th	Post-enu chemo (cycles)	OS	DSS	Dx to death (months)
121	M	OS	46	12	D	2b	2.5	pT1	0	Neg	Neg	
122	M	OS	121	21	E	3d	0.0	pT1	0	Neg	Neg	
123	M	OD	101	25	D	2b	0.8	pT1	0	Neg	Neg	
124	F	OS	76	20	D	2b	0.0	N/A	0	Neg	Neg	
125	M	OD	92	32	D	2b	0.0	pT1	0	Neg	Neg	
126	M	OS	122	36	E	3c	0.0	pT3b	6	Neg	Neg	
127	M	OD	165	20	E	3c	0.1	pT1	3	Neg	Neg	
128	M	OS	133	42	?	?	0.0	N/A	6	Neg	Neg	
129	M	OD	92	9	D	2b	0.1	pT1	0	Neg	Neg	
130	M	OS	18	32	D	2b	0.0	pT1	8	Neg	Neg	
131	F	OS	9	24	E	3d	0.0	N/A	0	Pos	Pos	9
132	F	OS	72	31	E	3b	0.0	pT3b	7	Neg	Neg	
133	F	OS	13	13	E	3d	0.2	pT1	0	Neg	Neg	
134	F	OS	137	15	D	2b	0.1	pT1	0	Neg	Neg	
135	F	OD	71	9	D	2b	0.1	pT2b	0	Neg	Neg	
136	F	OD	109	26	E	3b	0.2	pT1	0	Neg	Neg	
137	F	OS	100	40	E	3b	0.0	pT1	0	Neg	Neg	
138	M	OS	59	33	D	2b	0.0	pT1	2	Neg	Neg	
139	M	OS	76	12	D	2b	0.0	pT1	3	Neg	Neg	
140	M	OD	116	23	E	3d	0.0	pT1	0	Neg	Neg	
141	M	OD	47	26	E	3c	0.0	pT3b	6	Neg	Neg	
142	M	OD	92	4	?	?	0.0	N/A	0	Neg	Neg	
143	M	OS	104	26	E	3b	0.0	pT1	0	Neg	Neg	
144	F	OS	154	8	D	2b	0.0	pT1	0	Neg	Neg	
145	F	OS	73	8	D	2b	0.0	pT3c	4	Neg	Neg	
146	F	OD	138	28	E	3c	0.0	pT1	0	Neg	Neg	
147	M	OD	34	4	E	3c	0.4	pT2a	6	Neg	Neg	
148	F	OD	125	18	E	3c	0.0	pT3b	0	Neg	Neg	
149	M	OS	83	3	D	2b	2.7	pT1	0	Neg	Neg	
150	F	OS	160	20	E	3c	0.0	N/A	6	Neg	Neg	
151	F	OD	116	46	E	3c	0.0	pT1	0	Neg	Neg	
152	M	OD	158	4	E	3c	0.0	pT1	6	Neg	Neg	
153	F	OD	60	11	D	2b	0.3	pT1	2	Neg	Neg	
154	M	OS	102	13	E	3c	0.0	pT1	0	Neg	Neg	
155	F	OS	111	25	D	2b	0.0	pT3b	1	Neg	Neg	
156	M	OS	85	20	E	3b	0.0	pT4	6	Neg	Neg	
157	M	OD	114	16	E	3b	0.3	pT3b	6	Neg	Neg	
158	F	OS	48	24	D	2b	0.0	pT3b	3	Neg	Neg	
159	M	OD	106	15	E	3c	0.0	pT3b	2	Neg	Neg	
160	F	OD	130	17	E	3b	0.0	pT3b	4	Neg	Neg	
161	M	OD	76	46	?	?	0.0	N/A	6	Neg	Neg	
162	F	OD	161	25	E	3c	0.0	pT3b	9	Neg	Neg	
163	M	OD	38	31	E	3b	0.0	pT1	0	Neg	Neg	
164	M	OD	109	26	?	?	0.0	N/A	0	Neg	Neg	
165	F	OD	77	11	E	3b	0.4	pT1	0	Neg	Neg	
166	F	OD	64	38	E	3b	0.3	pT3b	7	Neg	Neg	
167	M	OS	123	8	E	3b	0.0	pT2a	3	Neg	Neg	
168	M	OS	72	26	E	3c	0.0	pT3b	7	Neg	Neg	
169	F	OD	97	19	E	3d	0.1	pT1	0	Neg	Neg	
170	F	OS	77	49	E	3c	0.3	pT3b	6	Neg	Neg	
171	F	OD	106	13	D	2b	0.9	pT1	0	Neg	Neg	
172	F	OS	94	16	D	2b	0.1	pT1	0	Neg	Neg	
173	M	OS	133	21	E	3d	0.3	pT3b	3	Neg	Neg	
174	F	OS	65	18	E	3c	0.0	pT1	2	Neg	Neg	
175	M	OS	58	18	E	3d	0.0	pT4	6	Neg	Neg	
176	M	OS	36	27	E	3b	0.1	pT3b	6	Neg	Neg	
177	M	OS	104	20	D	2b	0.1	pT1	0	Neg	Neg	
178	F	OS	137	32	E	3c	0.2	pT1	0	Neg	Neg	
179	M	OD	104	15	E	3c	0.0	pT1	0	Neg	Neg	
180	M	OS	84	23	E	3c	0.1	pT1	0	Neg	Neg	

ID	Sex	Laterality	FU (months)	Age at Dx	IIRC	cTNM	Dx to Enu	pTNM 8th	Post-enu chemo (cycles)	OS	DSS	Dx to death (months)
181	M	OD	124	34	E	3d	9.2	pT4	6	Neg	Neg	
182	F	OD	97	26	D	2b	0.0	pT1	0	Neg	Neg	
183	F	OS	16	10	E	3c	0.0	pT3b	6	Neg	Neg	
184	F	OD	139	37	E	3b	0.2	pT1	0	Neg	Neg	
185	F	OS	91	5	E	3b	0.0	pT1	0	Neg	Neg	
186	F	OD	108	43	E	3c	0.0	pT3b	6	Neg	Neg	
187	M	OD	95	10	E	3b	0.1	pT3b	6	Neg	Neg	
188	M	OS	90	34	E	3c	0.0	pT3b	3	Neg	Neg	
189	M	OD	25	29	E	3b	0.0	pT3b	6	Neg	Neg	
190	M	OD	103	40	E	3d	0.0	pT1	0	Neg	Neg	
191	F	OS	79	21	E	3b	0.0	pT1	0	Neg	Neg	
192	M	OD	116	81	E	3b	0.0	pT1	0	Neg	Neg	
193	M	OS	6	25	E	3c	0.0	pT4	1	Pos	Pos	6
194	F	OD	93	42	D	2b	0.2	pT1	0	Neg	Neg	
195	M	OD	35	4	E	3c	0.0	pT1	3	Neg	Neg	
196	M	OS	138	20	?	?	0.0	N/A	0	Neg	Neg	
197	M	OD	82	22	D	2b	0.2	pT1	0	Neg	Neg	
198	F	OD	47	22	E	3b	0.3	pT3d	4	Neg	Neg	
199	M	OD	65	30	E	3c	0.0	pT3b	3	Neg	Neg	
200	M	OS	54	35	D	2b	1.9	pT3b	6	Neg	Neg	
201	M	OD	130	15	E	3b	0.0	pT1	3	Neg	Neg	
202	F	OS	142	33	E	3b	0.0	pT1	3	Neg	Neg	
203	F	OS	70	41	D	2b	0.4	pT3a	3	Neg	Neg	
204	F	OS	100	28	E	3c	0.1	pT2a	0	Neg	Neg	
205	M	OD	149	10	E	3b	0.0	pT1	0	Neg	Neg	
206	M	OD	59	30	E	3b	0.0	pT3c	5	Neg	Neg	
207	F	OS	79	22	E	3d	0.6	pT1	3	Neg	Neg	
208	M	OS	62	50	D	2b	1.1	pT1	2	Neg	Neg	
209	F	OS	69	18	E	3d	0.0	pT1	0	Neg	Neg	
210	M	OD	64	23	E	3b	0.0	pT2b	4	Neg	Neg	
211	M	OD	101	18	D	2b	0.0	pT1	0	Neg	Neg	
212	M	OD	96	34	D	2b	0.0	pT1	0	Neg	Neg	
213	F	OD	144	38	E	3c	0.0	pT2a	3	Neg	Neg	
214	F	OD	67	25	E	3d	0.0	pT1	3	Neg	Neg	
215	F	OD	155	62	E	3d	0.0	pT1	3	Neg	Neg	
216	F	OS	104	54	E	3b	0.0	pT1	0	Neg	Neg	
217	M	OS	167	3	?	?	0.0	pT1	0	Neg	Neg	
218	M	OD	76	35	D	2b	0.0	pT1	6	Neg	Neg	
219	F	OS	83	54	E	3c	0.5	pT1	0	Neg	Neg	
220	F	OS	58	1	E	3d	0.0	pT1	0	Neg	Neg	
221	M	OS	11	41	E	3c	0.4	pT4	6	Pos	Pos	11
222	M	OD	99	42	E	3c	0.0	pT3b	6	Neg	Neg	
223	M	OD	62	12	E	3d	0.0	pT1	0	Neg	Neg	
224	M	OS	91	11	E	3b	0.2	pT1	0	Neg	Neg	
225	F	OS	103	29	E	3c	0.2	pT3d	6	Neg	Neg	
226	F	OS	110	24	D	2b	0.2	pT1	0	Neg	Neg	
227	M	OD	27	35	E	3c	0.2	pT4	12	Pos	Pos	27
228	M	OD	55	31	E	3c	0.3	pT1	0	Neg	Neg	
229	M	OD	142	26	E	3c	0.2	pT1	3	Neg	Neg	
230	M	OS	46	34	E	3b	0.0	pT3b	6	Neg	Neg	
231	F	OS	155	40	?	?	0.0	N/A	0	Neg	Neg	
232	F	OS	46	13	E	3b	0.3	pT1	0	Neg	Neg	
233	M	OS	102	39	D	2b	0.1	pT3b	6	Neg	Neg	
234	M	OS	143	88	E	3b	0.2	pT1	0	Neg	Neg	
235	M	OD	94	13	E	3d	0.0	pT3b	6	Neg	Neg	
236	M	OD	128	3	D	2b	0.0	pT2a	6	Neg	Neg	
237	M	OS	136	29	E	3b	0.2	pT1	0	Neg	Neg	
238	F	OS	145	18	E	3d	0.2	pT3a	5	Neg	Neg	
239	M	OS	70	37	D	2b	0.0	N/A	6	Neg	Neg	
240	F	OD	123	25	D	2b	0.1	pT1	0	Neg	Neg	

ID	Sex	Laterality	FU (months)	Age at Dx	IIRC	cTNM	Dx to Enu	pTNM 8th	Post-enu chemo (cycles)	OS	DSS	Dx to death (months)
241	M	OD	140	23	E	3c	0.0	pT2a	6	Neg	Neg	
242	F	OD	7	35	E	3c	0.0	pt3b	6	Pos	Pos	7
243	F	OS	86	28	?	?	0.0	pT3a	6	Neg	Neg	
244	M	OS	66	23	D	2b	0.3	pT3b	3	Neg	Neg	
245	F	OS	78	20	E	3b	0.0	pT1	0	Neg	Neg	
246	M	OS	116	22	D	2b	0.0	pT3b	3	Neg	Neg	
247	F	OD	120	12	E	3d	0.0	pT3b	3	Neg	Neg	
248	F	OD	98	37	D	2b	0.0	pT1	0	Neg	Neg	
249	M	OD	74	34	E	3d	0.0	pT3b	1	Neg	Neg	
250	F	OS	127	76	E	3d	0.1	pT2b	6	Neg	Neg	
251	M	OS	97	50	E	3d	0.0	pT1	0	Neg	Neg	
252	F	OD	156	33	E	3b	0.0	pT1	6	Neg	Neg	
253	F	OD	93	13	D	2b	0.1	pT1	0	Neg	Neg	
254	F	OS	115	7	E	3c	0.0	pT3b	6	Neg	Neg	
255	F	OS	60	41	D	2b	0.1	pT1	0	Neg	Neg	
256	F	OS	68	21	E	3d	0.1	pT1	2	Neg	Neg	
257	F	OD	111	11	D	2b	0.8	pT1	0	Neg	Neg	
258	M	OD	114	6	E	3d	0.0	pT4	6	Neg	Neg	
259	M	OD	54	4	E	3b	0.5	pT1	6	Neg	Neg	
260	M	OS	127	74	E	3b	0.2	pT1	5	Neg	Neg	
261	F	OS	23	13	E	3d	0.0	pT1	0	Neg	Neg	
262	M	OS	105	36	E	3c	0.0	pT1	0	Neg	Neg	
263	M	OS	119	10	E	3d	0.0	pT1	0	Neg	Neg	
264	F	OS	104	15	E	3d	0.2	pT3b	6	Neg	Neg	
265	F	OS	88	33	E	3b	0.0	pT1	0	Neg	Neg	
266	M	OS	50	24	E	3c	0.3	pT1	0	Neg	Neg	
267	M	OD	104	33	E	3c	0.0	pT3b	4	Neg	Neg	
268	F	OD	101	19	?	?	0.0	N/A	0	Neg	Neg	
269	M	OS	74	1	E	3d	0.5	pT1	0	Neg	Neg	
270	M	OS	13	28	E	3b	0.3	N/A	0	Neg	Neg	
271	M	OD	80	27	E	3c	0.0	N/A	6	Neg	Neg	
272	F	OS	86	17	E	3d	0.3	pT3b	8	Neg	Neg	
273	F	OS	97	51	D	2b	0.0	pT3b	6	Neg	Neg	
274	F	OS	55	49	D	2b	0.0	pT3c	0	Neg	Neg	
275	M	OS	83	14	E	3c	0.0	pT3a	4	Neg	Neg	
276	M	OD	14	33	E	3c	0.0	pT1	3	Neg	Neg	
277	F	OD	124	40	E	3b	0.1	pT1	0	Neg	Neg	
278	F	OS	124	50	E	3d	0.4	pT1	0	Neg	Neg	
279	F	OD	34	27	D	2b	0.2	pT1	1	Neg	Neg	
280	M	OD	130	21	E	3a	0.0	pT4	6	Neg	Neg	
281	M	OD	127	70	E	3b	0.2	pT1	0	Neg	Neg	
282	M	OS	104	2	E	3d	1.8	pT1	0	Neg	Neg	
283	M	OS	102	44	?	?	0.0	pT1	0	Neg	Neg	
284	F	OD	107	10	?	?	0.0	N/A	0	Neg	Neg	
285	F	OD	78	31	?	?	0.0	N/A	2	Neg	Neg	
286	F	OS	70	34	D	2b	0.0	pT1	0	Neg	Neg	
287	F	OS	83	23	?	?	0.0	Pt2a	6	Neg	Neg	
288	M	OD	10	24	E	3c	0.0	pT3d	6	Pos	Pos	10
289	M	OD	42	158	E	3b	4.0	pT2b	0	Neg	Neg	
290	F	OS	140	50	E	3b	0.0	pT1	3	Neg	Neg	
291	F	OS	124	8	E	3a	0.0	pT3b	4	Neg	Neg	
292	F	OS	115	20	D	2b	0.1	pT1	0	Neg	Neg	
293	M	OS	55	8	D	2b	0.5	pT1	0	Neg	Neg	
294	M	OD	36	22	E	3c	0.1	pT1	0	Neg	Neg	
295	M	OS	57	32	D	2b	0.2	pT1	1	Neg	Neg	
296	M	OD	74	43	E	3b	0.0	pT3b	6	Neg	Neg	
297	F	OS	87	21	E	3c	0.0	pT1	0	Neg	Neg	
298	M	OD	122	99	E	3c	0.0	pT1	0	Neg	Neg	
299	M	OS	67	50	D	2b	0.0	pT1	3	Neg	Neg	
300	F	OS	121	19	E	3d	0.0	pT3b	6	Neg	Neg	

ID	Sex	Laterality	FU (months)	Age at Dx	IIRC	cTNM	Dx to Enu	pTNM 8th	Post-enu chemo (cycles)	OS	DSS	Dx to death (months)
301	M	OD	31	13	?	?	0.0	N/A	0	Neg	Neg	
302	M	OS	62	17	E	3d	0.0	pT1	1	Neg	Neg	
303	M	OS	136	61	E	3d	0.0	pT2a	3	Neg	Neg	
304	M	OD	119	19	D	2b	0.0	pT2a	4	Neg	Neg	
305	F	OD	124	29	E	3c	0.0	pT3b	6	Neg	Neg	
306	M	OS	77	31	E	3c	0.0	pT3b	4	Neg	Neg	
307	F	OD	96	18	D	2b	0.2	pT1	0	Neg	Neg	
308	F	OS	120	26	D	2b	0.0	pT1	0	Neg	Neg	
309	F	OD	84	14	D	2b	0.1	pT1	0	Neg	Neg	
310	M	OD	21	24	E	3b	0.0	pT3b	4	Neg	Neg	
311	M	OD	100	29	E	3c	0.0	pT1	0	Neg	Neg	
312	F	OS	65	38	E	3d	0.2	pT1	4	Neg	Neg	
313	M	OS	107	29	E	3d	0.0	pT1	0	Neg	Neg	
314	F	OS	173	39	E	3c	0.0	pT1	7	Neg	Neg	
315	M	OD	123	21	?	?	0.0	pT1	0	Neg	Neg	
316	M	OS	118	30	E	3c	0.0	pT3b	6	Neg	Neg	
317	F	OS	66	19	E	3b	0.2	pT1	0	Neg	Neg	
318	F	OS	81	4	E	3d	0.0	N/A	0	Neg	Neg	
319	F	OS	129	8	E	3d	0.0	pT1	0	Neg	Neg	
320	F	OS	88	27	D	2b	0.0	pT1	0	Neg	Neg	
321	M	OD	125	27	D	2b	0.1	pT1	0	Neg	Neg	
322	M	OS	142	9	D	2b	0.3	pT3b	6	Neg	Neg	
323	F	OD	8	19	E	3c	0.2	pT3b	4	Pos	Pos	8
324	M	OS	102	13	D	2b	0.6	pT1	0	Neg	Neg	
325	M	OD	162	47	E	3b	0.0	pT1	3	Neg	Neg	
326	M	OS	100	25	E	3c	0.0	pT1	0	Neg	Neg	
327	F	OD	50	13	D	2b	0.0	pT3b	0	Neg	Neg	
328	M	OS	68	21	D	2b	0.0	pT1	0	Neg	Neg	
329	M	OD	132	22	D	2b	0.3	pT1	0	Neg	Neg	
330	F	OS	47	12	?	?	0.0	N/A	0	Neg	Neg	
331	M	OD	83	22	D	2b	0.5	pT1	0	Neg	Neg	
332	M	OD	156	21	E	3d	0.0	pT1	6	Neg	Neg	
333	M	OD	79	7	D	2b	0.0	N/A	0	Neg	Neg	
334	M	OD	80	49	D	2b	0.0	pT1	4	Neg	Neg	
335	M	OS	115	23	E	3c	0.2	pT1	6	Neg	Neg	
336	F	OD	74	19	D	2b	0.1	pT3b	0	Neg	Neg	
337	M	OS	99	45	D	2b	0.0	pT1	0	Neg	Neg	
338	M	OD	30	10	E	3d	0.5	pT3b	3	Neg	Neg	
339	F	OS	158	17	?	?	22.0	N/A	0	Neg	Neg	
340	F	OS	135	20	D	2b	0.0	pT3b	0	Neg	Neg	
341	F	OS	164	24	E	3b	0.0	pT1	3	Neg	Neg	
342	M	OS	123	30	E	3b	0.0	pT1	0	Neg	Neg	
343	M	OS	106	38	E	3b	0.1	pT2b	3	Neg	Neg	
344	M	OD	140	28	E	3c	0.2	pT1	0	Neg	Neg	
345	F	OS	123	23	E	3b	0.0	pT1	0	Neg	Neg	
346	M	OS	7	31	E	3b	0.0	pT3b	0	Pos	Pos	7
347	F	OS	107	25	E	3c	0.0	pT1	6	Neg	Neg	
348	F	OD	56	21	D	2b	0.2	pT1	0	Neg	Neg	
349	F	OD	45	23	E	3b	0.0	pT1	0	Neg	Neg	
350	F	OS	69	36	?	?	0.0	N/A	0	Neg	Neg	
351	M	OS	106	28	E	3b	0.0	pT2b	6	Neg	Neg	
352	M	OS	54	36	D	2b	0.3	pT3b	6	Neg	Neg	
353	M	OD	19	33	E	3c	0.1	pT3b	1	Neg	Neg	
354	M	OD	135	8	D	2b	1.0	pT3b	6	Neg	Neg	
355	F	OD	17	32	E	3c	0.0	pT4	3	Pos	Pos	17
356	F	OS	68	29	E	3d	1.2	pT3b	6	Neg	Neg	
357	M	OS	30	56	E	3b	0.0	pT3b	6	Neg	Neg	
358	M	OD	72	111	E	3b	0.0	pT3b	6	Neg	Neg	
359	F	OD	118	4	D	2b	0.2	pT3a	3	Neg	Neg	
360	M	OD	74	11	D	2b	0.2	N/A	5	Neg	Neg	

ID	Sex	Laterality	FU (months)	Age at Dx	IIRC	cTNM	Dx to Enu	pTNM 8th	Post-enu chemo (cycles)	OS	DSS	Dx to death (months)
361	M	OS	146	16	E	3c	0.5	pT1	3	Neg	Neg	
362	M	OS	153	23	E	3b	0.3	pT1	2	Neg	Neg	
363	M	OD	112	41	D	2b	0.1	pT1	2	Neg	Neg	
364	M	OS	168	71	E	3b	0.0	pT1	6	Neg	Neg	
365	M	OS	73	36	E	3c	0.1	pT1	0	Neg	Neg	
366	M	OS	125	5	E	3b	0.1	pT3b	6	Neg	Neg	
367	F	OS	96	43	D	2b	0.0	pT1	0	Neg	Neg	
368	F	OD	101	5	D	2b	0.0	pT1	0	Neg	Neg	
369	F	OS	89	11	D	2b	0.3	pT1	0	Neg	Neg	
370	F	OS	94	3	E	3d	0.2	pT1	0	Neg	Neg	
371	M	OD	103	2	E	3b	0.1	pT1	0	Neg	Neg	
372	M	OS	74	28	?	?	0.0	N/A	0	Neg	Neg	
373	M	OD	85	21	D	2b	0.0	pT1	0	Neg	Neg	
374	M	OS	108	22	D	2b	0.2	pT2a	6	Neg	Neg	
375	F	OS	90	12	D	2b	0.1	pT1	0	Neg	Neg	
376	M	OD	102	12	D	2b	0.0	pT1	0	Neg	Neg	
377	M	OD	91	61	E	3d	0.2	pT1	0	Neg	Neg	
378	M	OS	134	11	E	3b	0.0	pT1	0	Neg	Neg	
379	F	OD	139	44	E	3b	0.9	pT1	0	Neg	Neg	
380	M	OS	136	20	?	?	0.0	N/A	0	Neg	Neg	
381	M	OS	84	24	D	2b	0.0	pT1	0	Neg	Neg	
382	M	OD	104	7	D	2b	0.0	pT1	0	Neg	Neg	
383	M	OS	116	45	E	3b	0.1	pT1	0	Neg	Neg	
384	F	OD	73	19	E	3c	0.6	pT1	0	Neg	Neg	
385	F	OD	141	15	D	2b	0.5	pT1	2	Neg	Neg	
386	M	OD	143	20	E	3d	0.4	pT1	0	Neg	Neg	
387	F	OS	8	42	E	3c	0.0	pT2b	0	Pos	Pos	8
388	F	OS	135	18	E	3c	0.4	pT3b	2	Neg	Neg	
389	M	OD	81	3	D	2b	0.0	pT1	0	Neg	Neg	
390	M	OS	84	22	E	3b	0.0	pT1	0	Neg	Neg	
391	M	OS	107	24	E	3d	0.1	pT1	0	Neg	Neg	
392	F	OD	72	29	E	3c	0.4	pT1	0	Neg	Neg	
393	M	OD	6	63	E	3b	0.0	pT4	4	Pos	Pos	6
394	F	OD	116	16	E	3d	0.0	pT3a	2	Neg	Neg	
395	F	OD	111	13	E	3c	0.1	pT1	0	Neg	Neg	
396	M	OD	121	41	E	3b	0.0	pT4	7	Neg	Neg	
397	F	OD	58	20	E	3c	0.0	pT1	7	Neg	Neg	
398	F	OD	17	14	E	3c	0.0	pT2a	2	Neg	Neg	
399	F	OD	101	32	E	3d	0.1	pT1	0	Neg	Neg	
400	F	OS	131	77	E	3d	0.0	pT1	0	Neg	Neg	
401	M	OS	89	71	E	3b	0.1	pT1	0	Neg	Neg	
402	M	OS	66	27	?	?	0.0	N/A	6	Neg	Neg	
403	M	OD	71	33	D	2b	0.0	pT2a	0	Neg	Neg	
404	F	OS	88	35	D	2b	0.6	pT1	0	Neg	Neg	
405	M	OD	74	17	D	2b	1.3	pT1	0	Neg	Neg	
406	F	OS	65	21	D	2b	0.0	pT1	0	Neg	Neg	
407	F	OS	78	23	E	3b	0.0	pT3b	6	Neg	Neg	
408	F	OS	108	18	D	2b	0.0	N/A	7	Neg	Neg	
409	F	OD	13	19	E	3c	0.0	pT3b	3	Neg	Neg	
410	F	OD	112	18	E	3c	0.0	pT3b	0	Neg	Neg	
411	M	OS	12	39	?	?	0.1	N/A	0	Pos	Pos	12
412	F	OD	75	20	E	3c	0.8	pT3b	6	Neg	Neg	
413	F	OD	118	27	E	3b	0.2	pT1	0	Neg	Neg	
414	F	OD	134	2	E	3e	0.0	pT2a	0	Neg	Neg	
415	M	OS	106	44	E	3b	0.0	pT3b	6	Neg	Neg	
416	M	OS	18	29	E	3c	0.0	pT1	1	Neg	Neg	
417	M	OS	25	14	E	3c	0.1	pT1	1	Neg	Neg	
418	M	OD	134	43	E	3c	0.0	pT1	0	Neg	Neg	
419	F	OD	140	12	E	3c	0.8	pT1	6	Neg	Neg	
420	F	OD	105	11	D	2b	0.0	pT1	0	Neg	Neg	

ID	Sex	Laterality	FU (months)	Age at Dx	IIRC	cTNM	Dx to Enu	pTNM 8th	Post-enu chemo (cycles)	OS	DSS	Dx to death (months)
421	M	OS	119	8	D	2b	0.1	pT3b	6	Neg	Neg	
422	M	OS	130	27	D	2b	0.0	pT1	1	Neg	Neg	
423	F	OS	60	24	E	3d	0.4	pT1	0	Neg	Neg	
424	M	OD	66	18	E	3d	0.0	pT3d	6	Neg	Neg	
425	F	OS	71	17	?	?	0.0	N/A	5	Neg	Neg	
426	M	OS	74	22	E	3d	0.1	pT1	6	Neg	Neg	
427	M	OS	22	19	E	3c	0.4	pT1	0	Neg	Neg	
428	M	OD	121	40	E	3b	0.0	pT3a	6	Neg	Neg	
429	F	OD	110	16	E	3d	0.0	pT1	0	Neg	Neg	
430	F	OD	59	25	D	2b	0.0	N/A	10	Neg	Neg	
431	M	OS	100	50	E	3c	0.1	pT3b	6	Neg	Neg	
432	M	OD	121	21	E	3d	0.4	pT3b	0	Neg	Neg	
433	M	OD	11	27	E	3c	0.0	pT4	7	Pos	Pos	11
434	F	OS	44	89	E	3b	0.0	N/A	6	Neg	Neg	
435	M	OS	31	26	?	?	0.0	N/A	0	Neg	Neg	
436	M	OS	125	32	D	2b	1.2	pT2a	0	Neg	Neg	
437	M	OD	65	12	D	2b	0.0	pT1	0	Neg	Neg	
438	F	OD	121	31	E	3c	0.0	pT1	0	Neg	Neg	
439	M	OD	130	35	E	3b	0.0	pT3b	5	Neg	Neg	
440	F	OD	9	26	E	3c	0.0	pT3b	3	Pos	Pos	9
441	F	OD	120	42	E	3c	0.0	pT1	0	Neg	Neg	
442	M	OD	95	26	E	3c	0.0	pT1	0	Neg	Neg	
443	F	OD	133	41	E	3c	0.0	pT1	0	Neg	Neg	
444	M	OS	1	5	E	3c	0.0	pT1	1	Pos	Neg	1
445	M	OD	142	24	E	3c	0.0	pT1	0	Neg	Neg	
446	M	OD	87	18	E	3d	0.0	pT3b	6	Neg	Neg	
447	M	OS	93	65	D	2b	0.0	pT3b	5	Neg	Neg	
448	M	OD	135	20	D	2b	0.0	pT1	0	Neg	Neg	
449	M	OS	124	94	E	3b	0.1	pT2a	3	Neg	Neg	
450	M	OD	143	24	E	3b	0.3	pT1	3	Neg	Neg	
451	F	OS	20	73	E	3b	0.7	pT2b	9	Neg	Neg	
452	F	OD	112	66	D	2b	0.1	pT2b	4	Neg	Neg	
453	M	OD	27	79	D	2b	0.0	pT1	6	Neg	Neg	
454	F	OD	69	17	D	2b	1.2	pT1	0	Neg	Neg	
455	M	OS	142	4	E	3b	0.1	pT1	0	Neg	Neg	
456	M	OD	67	21	E	3b	0.0	pT3b	6	Neg	Neg	
457	M	OS	93	16	E	3d	0.0	pT3b	6	Neg	Neg	
458	F	OS	48	34	E	3d	0.0	pT4	0	Neg	Neg	
459	F	OS	118	13	E	3b	0.0	pT1	0	Neg	Neg	
460	F	OS	17	29	E	3c	8.0	pT3b	0	Pos	Pos	9
461	M	OD	103	13	E	3d	0.4	pT1	0	Neg	Neg	
462	M	OS	136	37	D	2b	0.0	pT1	0	Neg	Neg	
463	M	OS	63	20	E	3d	0.2	pT1	0	Neg	Neg	
464	F	OS	10	33	E	3c	0.1	pT4	3	Pos	Pos	9
465	M	OS	105	30	E	3d	0.0	pT4	6	Neg	Neg	
466	M	OS	16	16	E	3d	0.1	pT3b	6	Neg	Neg	
467	M	OS	98	35	D	2b	0.2	pT1	0	Neg	Neg	
468	F	OS	84	7	D	2b	0.0	pT1	0	Neg	Neg	
469	M	OS	98	51	D	2b	0.0	pT3b	6	Neg	Neg	
470	F	OS	136	19	E	3b	0.0	pT1	0	Neg	Neg	
471	F	OS	101	37	E	3c	0.0	pT1	0	Neg	Neg	
472	M	OD	122	38	E	3b	0.0	pT1	2	Neg	Neg	
473	F	OS	164	32	E	3b	0.1	pT3b	8	Neg	Neg	
474	M	OD	93	30	E	3d	0.2	pT2a	6	Neg	Neg	
475	F	OS	86	6	D	2b	0.0	pT2a	0	Neg	Neg	
476	M	OS	83	27	E	3b	0.0	pT1	0	Neg	Neg	
477	M	OD	91	63	E	3b	0.5	pT2b	6	Neg	Neg	
478	M	OS	123	28	D	2b	0.3	pT1	0	Neg	Neg	
479	M	OS	75	14	E	3d	2.0	pT1	3	Neg	Neg	
480	M	OD	114	35	D	2b	0.4	pT1	0	Neg	Neg	

ID	Sex	Laterality	FU (months)	Age at Dx	IIRC	cTNM	Dx to Enu	pTNM 8th	Post-enu chemo (cycles)	OS	DSS	Dx to death (months)
481	M	OD	142	59	E	3c	0.4	pT3b	5	Neg	Neg	
482	F	OD	90	22	D	2b	0.0	pT1	0	Neg	Neg	
483	F	OD	91	28	E	3c	0.0	pT4	6	Neg	Neg	
484	F	OS	111	31	E	3b	0.2	pT1	0	Neg	Neg	
485	F	OD	118	19	?	?	0.0	N/A	0	Neg	Neg	
486	F	OD	83	47	E	3b	1.3	pT2b	3	Neg	Neg	
487	M	OS	95	55	E	3c	0.0	N/A	0	Neg	Neg	
488	M	OS	136	41	E	3c	0.0	pT1	1	Neg	Neg	
489	F	OD	88	12	E	3c	0.0	pt3b	6	Neg	Neg	
490	F	OS	89	29	D	2b	0.2	pT1	0	Neg	Neg	
491	M	OD	92	18	D	2b	0.1	pT1	0	Neg	Neg	
492	M	OD	94	21	D	2b	0.0	pT1	0	Neg	Neg	
493	F	OD	132	43	E	3d	0.0	pT3b	2	Neg	Neg	
494	M	OS	114	41	D	2b	0.0	pt1	0	Neg	Neg	
495	M	OS	127	24	E	3c	0.0	pT3a	6	Neg	Neg	
496	M	OS	129	18	D	2b	0.0	pT1	0	Neg	Neg	
497	F	OD	34	9	D	2b	0.0	N/A	0	Neg	Neg	
498	F	OD	141	31	?	?	0.0	N/A	0	Neg	Neg	
499	M	OS	127	11	D	2b	0.0	pT1	6	Neg	Neg	
500	M	OD	71	28	E	3b	0.5	N/A	0	Neg	Neg	
501	F	OS	130	24	D	2b	0.0	pT3b	6	Neg	Neg	
502	F	OD	88	11	E	3d	0.0	pT1	8	Neg	Neg	
503	M	OS	96	33	D	2b	0.1	pT1	0	Neg	Neg	
504	M	OS	99	21	E	3d	0.2	pT2b	6	Neg	Neg	
505	M	OD	94	15	E	3c	0.1	pT2a	0	Neg	Neg	
506	M	OS	83	44	?	?	0.0	N/A	0	Neg	Neg	
507	M	OS	121	31	E	3b	0.0	pT1	0	Neg	Neg	
508	M	OS	128	11	E	3c	0.0	pT1	0	Neg	Neg	
509	M	OD	50	71	E	3d	0.0	pT2b	6	Neg	Neg	
510	M	OS	48	70	D	2b	0.1	pT2b	4	Neg	Neg	
511	F	OS	156	77	E	3c	0.2	pT2a	3	Neg	Neg	
512	F	OD	93	34	D	2b	0.3	pT1	0	Neg	Neg	
513	M	OD	83	8	E	3c	0.0	pT1	0	Neg	Neg	
514	M	OD	63	44	D	2b	0.0	pT1	0	Neg	Neg	
515	M	OS	97	14	E	3c	0.0	pT3a	6	Neg	Neg	
516	M	OD	79	35	D	2b	0.2	pT1	0	Neg	Neg	
517	F	OS	87	22	D	2b	0.4	pT1	0	Neg	Neg	
518	F	OS	51	47	D	2b	0.4	pT1	0	Neg	Neg	
519	F	OD	88	43	D	2b	0.2	pT1	0	Neg	Neg	
520	M	OD	58	6	D	2b	0.0	pT1	0	Neg	Neg	
521	F	OS	127	13	D	2b	0.0	pT1	0	Neg	Neg	
522	M	OD	84	89	D	2b	0.2	pT1	0	Neg	Neg	
523	M	OS	123	13	E	3c	0.0	pT1	0	Neg	Neg	
524	F	OS	112	16	D	2b	0.0	pT3b	6	Neg	Neg	
525	M	OS	71	17	E	3d	0.0	pT1	1	Neg	Neg	
526	M	OS	88	29	E	3b	0.0	pT3b	6	Neg	Neg	
527	M	OS	89	20	?	?	0.0	pT1	0	Neg	Neg	
528	M	OD	104	24	E	3b	0.0	pT3a	4	Neg	Neg	
529	M	OD	68	8	E	3c	0.0	N/A	6	Neg	Neg	
530	F	OD	112	29	E	3b	0.0	pT3b	6	Neg	Neg	
531	M	OD	20	3	E	3e	11.7	pT4	6	Pos	Pos	8
532	M	OS	64	51	D	2b	0.0	N/A	0	Neg	Neg	
533	F	OD	121	23	E	3c	0.1	pT1	0	Neg	Neg	
534	F	OD	114	212	?	?	0.0	pT2b	3	Neg	Neg	
535	F	OS	164	24	E	3b	0.0	pT1	6	Neg	Neg	
536	M	OS	80	21	D	2b	0.0	pT3b	0	Neg	Neg	
537	F	OS	121	5	E	3d	0.1	pT1	0	Neg	Neg	
538	F	OD	4	28	E	3c	0.0	pT4	0	Pos	Pos	4
539	M	OS	71	12	D	2b	0.0	pT1	0	Neg	Neg	
540	F	OD	90	2	E	3d	0.0	N/A	0	Neg	Neg	

ID	Sex	Laterality	FU (months)	Age at Dx	IIRC	cTNM	Dx to Enu	pTNM 8th	Post-enu chemo (cycles)	OS	DSS	Dx to death (months)
541	M	OS	159	11	E	3b	0.1	pT1	3	Neg	Neg	
542	M	OS	116	41	E	3d	0.1	pT3b	6	Neg	Neg	
543	M	OD	56	15	D	2b	0.0	pT1	0	Neg	Neg	
544	M	OD	73	23	D	2b	0.0	pT1	0	Neg	Neg	
545	M	OD	59	46	D	2b	0.4	pT1	0	Neg	Neg	
546	M	OS	93	19	?	?	0.0	pT1	0	Neg	Neg	
547	M	OD	124	52	E	3b	0.9	pT3b	0	Neg	Neg	
548	M	OS	28	38	E	3d	0.0	N/A	15	Neg	Neg	
549	M	OD	56	22	D	2b	0.0	pT1	2	Neg	Neg	
550	F	OD	61	22	E	3b	0.0	pT1	0	Neg	Neg	
551	M	OD	54	14	D	2b	0.0	N/A	0	Neg	Neg	
552	M	OS	61	38	E	3d	0.2	pT3b	6	Neg	Neg	
553	F	OS	76	55	E	3c	0.0	pT3a	6	Neg	Neg	
554	M	OD	74	18	E	3d	0.0	pT3d	6	Neg	Neg	
555	F	OS	75	11	E	3d	0.0	pT3b	2	Neg	Neg	
556	F	OS	146	24	E	3b	0.3	pT3b	6	Neg	Neg	
557	M	OS	87	54	D	2b	0.1	pT1	0	Neg	Neg	
558	M	OD	119	32	D	2b	0.0	pT1	0	Neg	Neg	
559	M	OS	127	22	D	2b	0.2	pT1	3	Neg	Neg	
560	F	OD	18	38	E	3c	0.0	pT4	12	Pos	Pos	18
561	M	OD	120	16	D	2b	0.0	pT1	0	Neg	Neg	
562	M	OD	117	27	E	3c	0.0	pT1	0	Neg	Neg	
563	M	OS	92	54	E	3b	0.1	pT2a	0	Neg	Neg	
564	M	OD	107	37	D	2b	0.0	pT1	0	Neg	Neg	
565	F	OD	70	16	D	2b	0.3	pT1	0	Neg	Neg	
566	M	OS	75	26	E	3b	0.0	pT1	0	Neg	Neg	
567	F	OD	70	21	D	2b	0.0	pT1	0	Neg	Neg	
568	F	OD	94	25	D	2b	0.2	pt1	0	Neg	Neg	
569	M	OD	162	32	E	3c	0.0	pT1	1	Neg	Neg	
570	F	OS	122	24	E	3b	0.0	pT1	0	Neg	Neg	
571	M	OD	75	55	?	?	0.3	N/A	4	Neg	Neg	
572	M	OD	25	32	E	3b	0.5	pT1	0	Neg	Neg	
573	M	OS	79	24	D	2b	0.1	pT1	0	Neg	Neg	
574	M	OS	157	44	E	3b	0.0	pT1	6	Neg	Neg	
575	M	OS	157	27	E	3c	0.0	pT1	3	Neg	Neg	
576	M	OS	117	43	E	3b	0.0	pT1	0	Neg	Neg	
577	F	OD	74	44	E	3b	0.5	pT1	0	Neg	Neg	
578	M	OS	59	8	D	2b	0.0	pT3a	6	Neg	Neg	
579	M	OD	88	18	?	?	0.0	N/A	0	Neg	Neg	
580	F	OS	20	28	E	3c	0.0	pT1	0	Neg	Neg	
581	M	OS	79	36	D	2b	0.0	pT3c	5	Neg	Neg	
582	M	OD	104	20	D	2b	0.1	pT1	0	Neg	Neg	
583	F	OD	116	38	E	3b	0.0	pT3b	6	Neg	Neg	
584	M	OS	94	30	D	2b	0.1	pT1	0	Neg	Neg	
585	F	OD	122	23	E	3c	0.0	pT2a	0	Neg	Neg	
586	M	OD	127	17	E	3c	0.0	pT1	0	Neg	Neg	
587	F	OS	132	18	E	3c	0.1	pT2a	5	Neg	Neg	
588	F	OS	110	16	E	3c	0.0	pT1	0	Neg	Neg	
589	M	OS	63	11	D	2b	0.0	pT1	0	Neg	Neg	
590	M	OD	117	50	E	3d	0.0	pT3b	4	Neg	Neg	
591	F	OS	106	57	E	3c	0.0	pT1	0	Neg	Neg	
592	M	OD	59	33	E	3b	0.0	pT2b	2	Neg	Neg	
593	F	OD	79	11	E	3b	0.0	pT1	0	Neg	Neg	
594	F	OD	136	19	D	2b	0.0	pT1	0	Neg	Neg	
595	F	OD	65	10	D	2b	0.0	pT2a	0	Neg	Neg	
596	F	OS	112	11	D	2b	0.2	pT1	0	Neg	Neg	
597	F	OD	14	33	E	3c	0.0	pT3b	3	Pos	Pos	14
598	F	OS	78	26	?	?	0.0	N/A	0	Neg	Neg	
599	F	OD	141	14	D	2b	0.4	pT2a	3	Neg	Neg	
600	F	OD	55	67	E	3d	0.3	pT1	0	Neg	Neg	

ID	Sex	Laterality	FU (months)	Age at Dx	IIRC	cTNM	Dx to Enu	pTNM 8th	Post-enu chemo (cycles)	OS	DSS	Dx to death (months)
601	M	OD	122	19	E	3b	0.0	pT1	3	Neg	Neg	
602	F	OS	58	17	?	?	0.0	N/A	0	Neg	Neg	
603	M	OD	86	4	E	3b	0.0	N/A	0	Neg	Neg	
604	F	OD	51	19	D	2b	0.3	pT1	0	Neg	Neg	
605	F	OS	99	12	D	2b	0.0	pT3a	6	Neg	Neg	
606	F	OS	41	34	E	3d	0.6	pT3b	6	Neg	Neg	
607	F	OD	160	3	?	?	4.5	pT3b	0	Neg	Neg	
608	F	OD	38	53	E	3b	0.0	pT3b	3	Neg	Neg	
609	M	OS	49	25	D	2b	0.0	pT1	3	Neg	Neg	
610	M	OS	90	28	D	2b	0.1	pT3a	3	Neg	Neg	
611	M	OS	106	11	E	3b	0.0	pT2a	0	Neg	Neg	
612	M	OS	29	15	D	2b	18.1	pT4	7	Pos	Pos	11
613	M	OS	144	21	E	3b	0.1	pT2a	0	Neg	Neg	
614	F	OS	112	21	D	2b	0.6	pT1	0	Neg	Neg	
615	M	OD	94	18	D	2b	0.0	pT1	0	Neg	Neg	
616	M	OD	84	2	E	3b	0.0	pT1	0	Neg	Neg	
617	F	OS	114	16	E	3d	0.0	pT1	0	Neg	Neg	
618	M	OD	52	14	E	3b	0.0	pT2a	3	Neg	Neg	
619	F	OD	70	12	D	2b	0.0	N/A	0	Neg	Neg	
620	M	OD	69	16	E	3d	0.0	pT1	0	Neg	Neg	
621	M	OS	48	20	?	?	0.0	pT1	0	Neg	Neg	
622	M	OD	128	31	D	2b	0.0	pT1	0	Neg	Neg	
623	F	OS	108	19	E	3c	0.1	pT3b	6	Neg	Neg	
624	F	OD	81	7	?	?	0.0	pT1	1	Neg	Neg	
625	M	OS	49	18	E	3c	0.2	pT1	0	Neg	Neg	
626	M	OS	71	24	D	2b	0.1	pT1	0	Neg	Neg	
627	M	OD	87	25	D	2b	0.0	pT1	0	Neg	Neg	
628	M	OS	16	10	E	3c	0.0	pT1	0	Neg	Neg	
629	F	OS	81	30	D	2b	0.0	N/A	0	Neg	Neg	
630	F	OD	13	23	E	3d	0.2	pT3b	0	Neg	Neg	
631	F	OS	129	17	E	3c	0.0	pT1	0	Neg	Neg	
632	M	OD	138	25	?	?	0.0	N/A	6	Neg	Neg	
633	M	OS	75	15	E	3c	0.6	pT1	0	Neg	Neg	
634	F	OD	56	84	E	3c	0.7	pT1	0	Neg	Neg	
635	M	OD	82	25	?	?	0.0	N/A	0	Neg	Neg	
636	F	OD	102	39	D	2b	0.0	pT1	0	Neg	Neg	
637	M	OD	105	16	D	2b	0.1	pT1	0	Neg	Neg	
638	M	OS	117	27	E	3c	0.0	pT1	0	Neg	Neg	
639	F	OD	121	20	E	3c	0.1	pT1	0	Neg	Neg	
640	M	OD	108	29	E	3b	0.0	pT1	0	Neg	Neg	
641	M	OD	54	24	E	3d	0.2	pT3b	4	Neg	Neg	
642	M	OD	104	22	D	2b	0.2	pT1	0	Neg	Neg	
643	M	OS	92	13	E	3d	0.1	pT3c	4	Neg	Neg	
644	F	OD	128	17	?	?	0.0	pT2b	6	Neg	Neg	
645	F	OD	131	56	D	2b	0.0	pT1	2	Neg	Neg	
646	F	OS	65	56	E	3c	0.0	pT1	6	Neg	Neg	
647	M	OS	106	27	E	3c	0.0	pT1	0	Neg	Neg	
648	M	OD	129	40	E	3c	0.0	pT3b	3	Neg	Neg	
649	M	OD	61	54	?	?	0.0	N/A	6	Neg	Neg	
650	M	OD	77	10	D	2b	0.0	pT3c	6	Neg	Neg	
651	F	OD	85	44	E	3d	0.2	pT2b	3	Neg	Neg	
652	M	OS	121	20	D	2b	0.0	pT1	0	Neg	Neg	
653	F	OD	81	34	D	2b	0.0	pT3b	5	Neg	Neg	
654	M	OS	61	16	E	3d	0.0	pT1	0	Neg	Neg	
655	M	OD	155	11	?	?	0.0	N/A	3	Neg	Neg	
656	F	OS	92	24	D	2b	0.5	pT1	0	Neg	Neg	
657	F	OD	82	32	E	3c	0.1	pT3b	6	Neg	Neg	
658	F	OD	70	21	E	3b	0.1	pT1	0	Neg	Neg	
659	M	OS	81	25	?	?	0.0	N/A	0	Neg	Neg	
660	F	OD	97	28	D	2b	0.0	pT1	0	Neg	Neg	

ID	Sex	Laterality	FU (months)	Age at Dx	IIRC	cTNM	Dx to Enu	pTNM 8th	Post-enu chemo (cycles)	OS	DSS	Dx to death (months)
661	M	OS	102	30	D	2b	0.0	pT1	0	Neg	Neg	
662	M	OD	65	17	E	3c	0.3	pT3b	0	Neg	Neg	
663	M	OD	128	4	D	2b	0.0	pT3b	6	Neg	Neg	
664	F	OD	84	23	E	3d	0.0	pT3b	7	Neg	Neg	
665	M	OS	38	22	D	2b	0.0	N/A	0	Neg	Neg	
666	M	OD	18	22	E	3b	0.0	pT1	0	Neg	Neg	
667	M	OD	14	10	D	2b	0.0	pT3b	2	Pos	Pos	14
668	M	OD	112	11	E	3c	0.0	pT3b	0	Neg	Neg	
669	M	OD	112	3	D	2b	3.0	pT3b	4	Neg	Neg	
670	F	OD	108	47	?	?	0.0	N/A	1	Neg	Neg	
671	M	OS	65	42	E	3c	0.0	pT1	0	Neg	Neg	
672	M	OS	57	40	D	2b	1.2	pT3a	6	Neg	Neg	
673	M	OD	84	11	D	2b	0.0	pT1	0	Neg	Neg	
674	F	OS	22	9	E	3c	0.0	pT1	3	Neg	Neg	
675	M	OD	95	32	D	2b	0.0	pT1	0	Neg	Neg	
676	F	OS	75	7	E	3b	0.3	pT1	0	Neg	Neg	
677	F	OS	97	15	E	3c	0.0	pT3b	6	Neg	Neg	
678	M	OS	123	59	E	3b	0.0	pT1	0	Neg	Neg	
679	M	OS	146	8	D	2b	0.5	pT1	3	Neg	Neg	
680	F	OD	143	51	D	2b	0.0	pT1	3	Neg	Neg	
681	M	OS	99	9	E	3c	0.0	pT3b	6	Neg	Neg	
682	F	OD	82	25	D	2b	0.0	N/A	6	Neg	Neg	
683	M	OS	94	15	E	3b	0.5	pT2b	3	Neg	Neg	
684	M	OD	11	23	E	3c	0.0	pT3b	5	Pos	Pos	11
685	M	OD	125	21	E	3c	0.2	pT3b	4	Neg	Neg	
686	F	OD	111	18	E	3b	0.1	pT3b	0	Neg	Neg	
687	F	OD	76	28	?	?	0.0	N/A	0	Neg	Neg	
688	M	OD	166	24	D	2b	0.0	pT1	3	Neg	Neg	
689	M	OS	92	11	D	2b	0.1	pT2a	0	Neg	Neg	
690	F	OD	104	29	E	3d	0.0	pT3b	0	Neg	Neg	
691	F	OD	47	9	E	3c	0.0	pT1	0	Neg	Neg	
692	M	OD	138	25	E	3c	0.0	pT2a	3	Neg	Neg	
693	M	OD	141	24	?	?	0.4	pT1	0	Neg	Neg	
694	M	OD	106	1	D	2b	0.0	pT3b	8	Neg	Neg	
695	F	OD	96	5	D	2b	0.0	N/A	0	Neg	Neg	
696	M	OD	10	22	E	3c	0.9	pT4	6	Pos	Pos	9
697	F	OD	104	37	?	?	0.0	N/A	0	Neg	Neg	
698	M	OD	100	34	D	2b	0.1	pT1	0	Neg	Neg	
699	F	OD	89	23	E	3d	0.0	pT1	0	Neg	Neg	
700	M	OD	133	15	D	2b	0.0	pT1	0	Neg	Neg	
